# Chemical Inhibitors and microRNAs (miRNA) Targeting the Mammalian Target of Rapamycin (mTOR) Pathway: Potential for Novel Anticancer Therapeutics

**DOI:** 10.3390/ijms14023874

**Published:** 2013-02-13

**Authors:** Naif AlQurashi, Saeed M. Hashimi, Ming Q. Wei

**Affiliations:** 1 Division of Molecular and Gene Therapies, Griffith Health Institute, School of Medical Science, Griffith University, Gold Coast, QLD 4215, Australia; 2 Biology Department, College of Medicine, University of Dammam, Dammam 31451, Saudi Arabia; E-Mail: nalqurashi@ud.edu.sa

**Keywords:** mTOR, Akt, S6K, Rapamycin, cancer, therapy, miRNA

## Abstract

The mammalian target of rapamycin (mTOR) is a critical regulator of many fundamental features in response to upstream cellular signals, such as growth factors, energy, stress and nutrients, controlling cell growth, proliferation and metabolism through two complexes, mTORC1 and mTORC2. Dysregulation of mTOR signalling often occurs in a variety of human malignant diseases making it a crucial and validated target in the treatment of cancer. Tumour cells have shown high susceptibility to mTOR inhibitors. Rapamycin and its derivatives (rapalogs) have been tested in clinical trials in several tumour types and found to be effective as anticancer agents in patients with advanced cancers. To block mTOR function, they form a complex with FKBP12 and then bind the FRB domain of mTOR. Furthermore, a new generation of mTOR inhibitors targeting ATP-binding in the catalytic site of mTOR showed potent and more selective inhibition. More recently, microRNAs (miRNA) have emerged as modulators of biological pathways that are essential in cancer initiation, development and progression. Evidence collected to date shows that miRNAs may function as tumour suppressors or oncogenes in several human neoplasms. The mTOR pathway is a promising target by miRNAs for anticancer therapy. Extensive studies have indicated that regulation of the mTOR pathway by miRNAs plays a major role in cancer progression, indicating a novel way to investigate the tumorigenesis and therapy of cancer. Here, we summarize current findings of the role of mTOR inhibitors and miRNAs in carcinogenesis through targeting mTOR signalling pathways and determine their potential as novel anti-cancer therapeutics.

## 1. Introduction

Mammalian target of rapamycin (mTOR) is a downstream effector of the PI3K/AKT pathway and the catalytic subunit of two biochemically distinct complexes, called mTORC1 and mTORC2. Each complex has its own protein composition, which reflects their differences in upstream signal integration, substrate regulation and biological process control in the pathway. They respond to a variety of stimuli, including growth factors, genotoxic stress, energy status, oxygen and amino acids. mTORC1 promotes anabolism, such as protein synthesis and lipid biogenesis, cell growth and cell cycle progression. At the same time, mTORC1 inhibits catabolism by blocking autophagy. In contrast, mTORC2 regulates cell survival, cell proliferation and metabolism. mTOR signalling is often dysregulated during various human cancers, driving most tumorigenesis from mutations of negative mTOR regulators or by oncogenic mutations. Thus, it has attracted great scientific and clinical interest in developing inhibitors that specifically target mTOR. Rapamycin and its derivatives (rapalogs), which inhibit mTOR function by a kinase-independent mechanism, have been tested in clinical trials in several tumour types. Rapalogs are selective for mTORC1 and effective as anticancer agents in patients with advanced renal cell carcinoma and mantle cell lymphoma. Recently, a new generation of mTOR inhibitors, which were designed to compete with ATP in the catalytic site of mTOR and inhibit both mTORC1 and mTORC2, showed potent and selective inhibition at nanomolar concentrations. Furthermore, growing evidence links miRNA deregulation of mTOR pathways to carcinogenesis in several human neoplasms, and their molecular signatures of deregulation have been associated with clinical features of several cancers. Downregulation of miRNAs were noted in some cancers, such as hepatocellular carcinomas, suggesting that tactics to regulate mTOR by elevating levels of miRNAs may be alternative therapeutic strategies for the treatment of cancer. The purpose of this review is to understand the role of mTOR inhibitors and miRNAs in carcinogenesis through targeting the mTOR signalling pathway and to determine whether they could have potential to be developed into novel therapies for a number of cancer types.

## 2. mTOR

Identification and cloning of mTOR, also known as FKBP12-rapamycin associated protein (FRAP), rapamycin and FKBP12 target (RAFT) or rapamycin target (RAPT), was completed shortly after the discovery of the two yeast genes, TOR1 and TOR2, in the budding yeast *Saccharomyces cerevisiae* by mutations that confer resistance to rapamycin (a naturally produced macrolide antibiotic) [1–4]. Rapamycin was isolated from a fungus (*Streptomyces hygroscopicus*) in a soil sample collected in Rapa Nui (known as Easter Island) in 1975 [5]. It forms a complex with FK506-binding protein (FKBP12) in mammals to inhibit mTOR [6].

**Figure 1:**
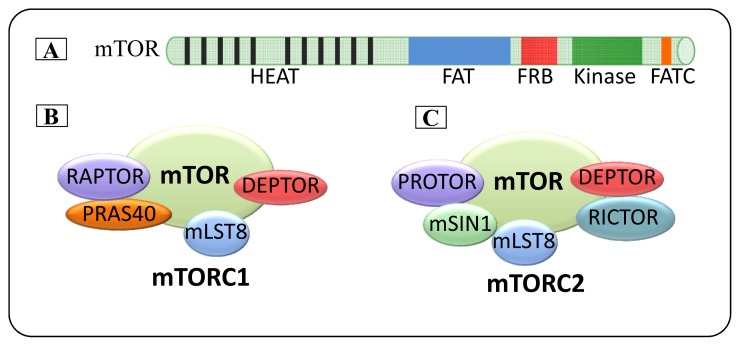
Schematic representation of the mTOR domain, mTORC1 and mTORC2 complexes. (**A**) The domain structure of mTOR is composed of HEAT repeats and FAT followed by FRB (a unique feature of mTOR that serves as the binding site for the inhibitory FKBP12 rapamycin complex), a kinase domain and the FATC (FAT C-terminus) domain. (**B**,**C**) mTOR complex 1 (mTORC1) and mTORC2 consist of shared and unique components. mTOR, mLST8 and DEPTOR are shared between the two complexes whereas RAPTOR and PRAS40 are unique to mTORC1. RICTOR, mSIN1 and PROTOR are unique to mTORC2.

mTOR is a member of the PI3K-related kinase (PIKK) family characterised by a high molecular weight (mTOR ~289 kDa) and contains 2549 amino acids, with 42% and 45% sequence identity to yeast TOR1 and TOR2, respectively (Figure 1A) [7–10]. The carboxy-terminal of mTOR contains the kinase domain, with significant homology to the PI3K lipid kinase domain [8]. However, evidence supports a role for TOR as a serine/threonine protein kinase [11–13]. The amino-terminal region contains 20 HEAT (Huntington, elongation factor 3, subunit of protein phosphatase 2A and TOR 1) repeat domains that are composed of two α helices of ~40 amino acids, each with a specific pattern of hydrophobic and hydrophilic residues [8]. HEAT repeats are known to mediate protein-protein interactions [14].

Other characteristic structures of mTOR include a FAT (FRAP, Ataxia telangiectasia mutated (ATM), Transformation/transcription domain associated protein (TRRAP) domain [15]. The carboxy-terminal end contains the FRB domain that binds the FKBP12-rapamycin complex followed by the catalytic domain that shows homology to phosphatidylinositol kinases and finally a FAT-C (FAT c-terminal) domain at the extreme carboxy-terminal of the protein [2,8,16,17].

### 2.1. mTOR Complexes

In mammalian cells mTOR exists in two large physically and functionally distinct signalling complexes, mTORC1 and mTORC2 [18,19].

#### 2.1.1. mTORC1

mTORC1 consists of mTOR as the catalytic subunit of the complex and four accessory proteins. Raptor (regulatory-associated protein of mTOR) physically links mTOR to its effectors, binding substrates and regulators (Figure 1B) [20–23]. mLST8 (mammalian lethal with SEC13 protein 8) mediates protein-protein interactions [24]. However, deletion of this protein does not affect mTORC1 activity *in vivo*, thus the function of mLST8 in mTORC1 is unclear [25]. PRAS40 (proline-rich AKT substrate 40 kDa, also known as AKT1S1) has been characterized as a negative regulator of mTORC1, which regulates mTORC1 kinase activity as a direct inhibitor of substrate binding [26–28]. Deptor (DEP-domain-containing mTOR-interacting protein) functions as a negative regulator of mTORC1 [29].

#### 2.1.2. mTORC2

mTORC2 consists of a complex that includes mTOR, Rictor (rapamycin-insensitive companion of mTOR) [30] and mSIN1 (mammalian stress-activated protein kinase interacting protein, also known as MAPKAP1) (Figure 1C) [31,32]. Rictor plays an important role in the phosphorylation of Akt [33]. PROTOR1 (protein observed with rictor-1) appears to help complex assembly [34,35]. Rictor, mSIN1 and PROTOR1 proteins are unique components of mTORC2, whereas the other known mTORC2 accessory proteins, including mLST8 and Deptor, are shared components with mTORC1. However, unlike mTORC1, mLST8 is essential for mTORC2 function, as deletion of this protein can severely reduce the stability and function of this complex [25]. Similar to its role in mTORC1, Deptor acts as a negative regulator of mTORC2 [29].

## 3. mTOR Regulation and Signalling

### 3.1. Upstream Regulators of mTORC1

**Figure 2:**
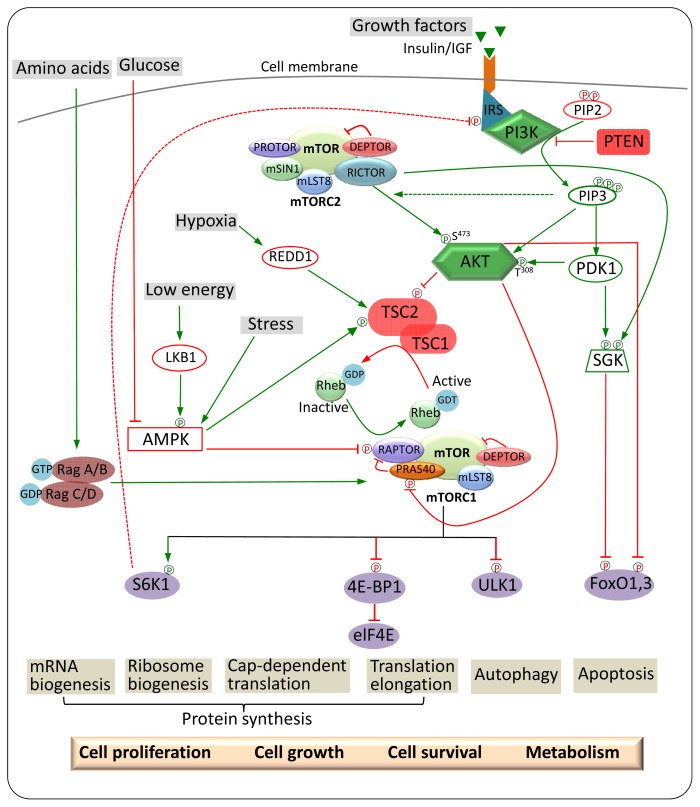
mTOR signalling pathway. mTORC1 activates S6K and inhibits 4E-BP in response to extracellular (growth factors, amino acids, glucose and cellular conditions) and intracellular signals (PI3K, AKT, PDK1, TSC1/2, AMPK, Rheb and Rag GTPases), resulting in activation of protein synthesis and suppression of autophagy. mTORC2 regulates apoptosis by phosphorylation of Akt at Ser473 and SGK. Green arrows represent activation, whereas the red represent inhibition; the green P represents activation through phosphorylation, whereas the red P represents inhibition through phosphorylation. Broken red lines indicate indirect inhibition.

mTORC1 regulates cell growth in response to four major regulatory inputs: nutrients, growth factors, energy and stress of the cell (Figure 2).

#### 3.1.1. Nutrients

Early studies have found that specific amino acids are crucial for mTORC1 signalling. Leucine was found to be an essential amino acid required for mTORC1 activation [36–40]. Recently, Nicklin and colleagues identified a mechanism by which Solute carrier family 7 member 5 (SLC7A5)/SLC3A2 regulates the exchange of L-glutamine out of cells and transport of L-leucine into cells, allowing intracellular leucine to play its role in mTORC1 signalling [37]. Several studies proposed that kinase mitogen-activated protein kinase kinase 3 (MAP4K3) and human vacuolar protein-sorting associated protein 34 (VPS34) are involved in nutrient sensing [41–44]. However, these mechanisms of sensing need more clarification. Other studies have proposed that TSC1 and TSC2 activate mTORC1 by amino acids [45]. However, the mTORC1 pathway in cells that lack either TSC1 or TSC2 remains sensitive to amino acid deprivation [43,46]. Recent work has shown that the Rag proteins, a family of four related small GTPases, have a strong link between amino acids and mTORC1 [47–49]. Active Rag interacts with RAPTOR in an amino acid-dependent manner, leading mTORC1 to co-localise with Rheb (Ras homologue enriched in brain) on the surface of late endosomes and lysosomes [47,48,50].

#### 3.1.2. Growth Factors

The mTORC1 pathway is stimulated by growth factors, such as insulin and insulin-like growth factors (IGFs), which bind to their receptor tyrosine kinases (e.g., IR/IGF-1R) and G protein receptors to promote translation, leading to cell growth and proliferation [51]. The stimulation of these pathways activates PI3K, which phosphorylates Akt, a downstream effector kinase of PI3K, which in turn phosphorylates TSC2 (Figure 2) [52,53]. TSC1 and TSC2 form a tight heterodimeric complex in which both negatively regulate mTORC1 signalling and act as a GTPase activating protein (GAP) for Rheb [54,55]. Rheb has an essential role in activation of mTORC1 by growth factors, which induce the GTP loading of Rheb, enabling it to bind to the kinase domain and activate mTORC1 in a GTP-dependent manner [35,56]. Additionally, Akt phosphorylates PRAS40, a novel growth factor-sensitive repressor of mTORC1, in a TSC1/2-independent manner, resulting in the dissociation of PRAS40 from mTORC1 [26,27]. Growth factors inhibit function of PRAS40 via activation of Akt, which phosphorylates the C-terminal Thr-246 residue in PRAS40, resulting in mTORC1 derepression [26]. Alternatively, growth factors can signal mTORC1 through other pathways, such as the RAS-ERK-RSK (Extracellular signal-Regulated Kinase) (ribosomal S6 kinase) pathway, which has a role in regulating transcription [57–59]. ERK phosphorylates TSC2 on Ser-664 *in vitro* and *in vivo*, which leads to TSC1/TSC2 complex disassembly and derepression of mTORC1 signalling [57]. RSK phosphorylates Ser-939, Thr-1462 and Ser-1798 residues on TSC2, which represses GAP activity of TSC2 [59]. Collectively, these findings suggest that RAS-ERK–RSK pathways, in parallel with the PI3K-AKT pathway, contain several inputs to activate mTORC1 signalling.

#### 3.1.3. Cellular Energy Status and Stress

Ribosomal biogenesis and mRNA translation, two downstream targets of mTORC1, consume high levels of cellular energy, indicating that mTORC1 senses cellular energy [60]. Recent studies reveal that the energy status of the cell is sensed by mTORC1 through AMP-activated protein kinase (AMPK) [61]. When the AMP:ATP ratio increases, AMPK is activated and phosphorylates TSC2, causing an increase in TSC2 GAP activity towards Rheb and subsequent inhibition of mTORC1 signalling [62,63]. Moreover, AMPK can directly phosphorylate raptor, resulting in the inhibition of mTORC1 signalling [64].

mTORC1 has been demonstrated to play a role in the response to numerous stress signals, such as hypoxia and DNA damage. In the case of hypoxia (low oxygen conditions), mTOR1 signalling is inhibited via the AMPK pathway by inducing the expression of REDD1 [65]. REDD1 negatively regulates mTORC1 by acting downstream of Akt and upstream of TSC1-TSC2 [66]. DNA damage is another stress signal that down-regulates mTOR signalling by p53 through the AMPK-TSC2 signalling pathway [67]. It was recently proposed that transcriptional targets of p53, sestrin 1 and sestrin 2, activate AMPK upon DNA damage [68].

### 3.2. Upstream and Downstream Regulators of mTORC2

Little is known about the upstream regulators and downstream effectors of mTORC2. It was proposed that growth factors play a direct and indirect role in mTORC2 regulation [69]. In *in vitro* studies, Sarbassov and his team demonstrated that insulin stimulates phosphorylation of Ser 473 in AKT at the cell membrane through the binding of PtdIns(3,4,5)P3 to its PH domain (pleckstrin homology) [69]. It has been proposed that mTORC2 plays important roles in proliferation, cell survival and metabolism, because of its activation by AKT [70]. Complete activation of AKT requires phosphorylation on Thr 308 and Ser 473 sites [71]. Phosphoinositide-dependent kinase 1(PDK1) and mTORC2 are responsible for phosphorylation of AKT Thr 308 and Ser 473, respectively [69]. Therefore, mTORC2 acts as a positive regulator for Akt. As a result of AKT inhibition by mTORC2 depletion, transcription factors, such as the forkhead box protein O1 (FoxO1) and FoxO3a, are activated because of the reduction of AKT phosphorylation [72]. FoxO1 and FoxO3a are involved in biological processes, such as stress resistance, metabolism, cell-cycle arrest and apoptosis [73]. Recent studies show that SGK1 (serum- and glucocorticoid-induced protein kinase 1), a member of the AGC family of protein kinases, is regulated by mTORC2, suggesting that SGK1 may also play important roles in regulating cellular proliferation [74]. Similar to AKT, SGK1 phosphorylates FoxO1 and FoxO3a, supporting the idea that the inhibition of phosphorylation of FoxO1 and FoxO3a is the result of deficient SGK1 activity in mTORC2-deficient cells [74]. Moreover, mTORC2 plays a role in a number of cellular processes, including cellular structure and motility, via regulation of protein kinase C (PKC) [30]. Knock-down mTORC2 components affect PKC-α phosphorylation and stability indirectly [30,75].

## 4. mTOR Signalling Pathways in Cancer

Elevated mTORC1 signalling has been detected in a large percentage of the most common human cancers [76]. mTOR drives most tumorigenesis from mutations of negative mTOR regulators, such as TSC1/TSC2, LKB1 and PTEN, or by oncogenic mutations, like PI3K and Akt [35,77]. The P13K-Akt-ERK pathways upstream of mTORC1 are activated downstream of both receptor tyrosine kinases (RTKs) and Ras [78,79]. Amplification and mutations of RTKs, such as Her2/neu, c-MET and EGFR, are examples in some common malignant tumours that lead to ligand-independent signalling from upstream RTKs [80]. Ras is a common oncogene in human cancers, which activates the PI3K-Akt pathway by inhibiting tumour suppressor NF1 [81]. Furthermore, in some cancers, mutated PI3K leads to the growth factor-independent activation of Akt. ERK is also activated in a variety of cancers by BRAF deregulation [76].

### 4.1. Downstream Targets of mTORC1 in Cancer

#### 4.1.1. 4E-BPs

4E-BPs are the major downstream targets of mTORC1 and are key regulators by which mTORC1 signalling contributes to tumorigenesis. 4E-BP1 negatively regulates the eIF4F complex, which drives mRNA translation initiation [82]. mTORC1 mediated phosphorylation of 4E-BP1 activates eIF4E, which leads to increased translation of mRNAs for pro-tumorigenic genes. As a result of this, inhibition of 4E-BP1 by phosphorylation has been identified in human cancers, such as breast, prostate and ovarian cancers [83–85]. In addition, an *in vivo* study has shown that eIF4E promoted cell survival in lymphoma by activating the translation of the anti-apoptotic protein myeloid leukaemia cell differentiation [86,87]. Consistent with this data, upregulation of eIF4E develops angiosarcomas, lung adenocarcinomas and hepatocellular adenomas in mice [88]. Furthermore, loss of 4E-BP1 and 4E-BP2 functions in mice increased tumorigenesis caused by inactivation of p53 [89]. In humans, over-expressing eIF4E levels have been observed in breast, colon, lung, prostate and bladder tumours [90–92]. This data highlights the importance of 4E-BP1 as a prognostic indicator in human cancer and eIF4E as a major target for anti-cancer therapies [93].

#### 4.1.2. S6 Kinases

The second major downstream target of mTORC1 that plays an important role in mTORC1 signalling and tumorigenesis is S6 kinase. The S6 kinases are regulators of protein synthesis, cell growth [94], S6 phosphorylation and signal transduction. mTORC1 phosphorylates S6K1 leading to increased mRNA translation. Upregulation of S6K1 in ovarian cancer cells increases cell growth and induces invasive and migratory phenotypes [95,96]. Activation of S6K1 has been reported in breast cancer cells and a number of other cancer cell lines as well [97–99]. In humans, phosphorylation of S6K1 associates with a higher cancer grade in patients with ovarian cancer, and the level of S6K1 is increased in primary breast tumours [97,100–102].

### 4.2. Downstream Targets of mTORC2 in Cancer

mTORC2 is responsible for phosphorylating and activating AKT, which may drive tumorigenesis. Since AKT is involved in the regulation of cell survival, inactivation of the tumour suppressor PTEN or mutations in PI3K may be dependent on the pro-survival activities of Akt [35,103]. Studies carried on cultured cells and mice reported that Rictor (a unique mTORC2 component) is required for the growth of tumour cells and prostate tumours deficient in PTEN, respectively [104–106]. Prostate epithelial cells lacking PTEN require mTORC2 to induce invasive prostate cancer. However, deleting Rictor protects PTEN heterozygous mice from prostate cancer [104]. Deletion of Rictor alone in the prostate epithelium is nonessential for the development of a normal prostate epithelium, suggesting that mTORC2 activity is necessary for PTEN-deficient cancer and not for normal prostate function [104]. Therefore, in this context, development of mTORC2 inhibitors as anti-cancer therapies might be useful. To date, probably the most effective way to target mTORC2 is by the recently developed ATP-competitive tyrosine-kinase inhibitors and by targeting PI3K. However, both strategies are considered mTORC2-nonspecific.

## 5. The Impact of mTOR Inhibitors in Cancer

### 5.1. Rapamycin and Rapalogs

**Figure 3:**
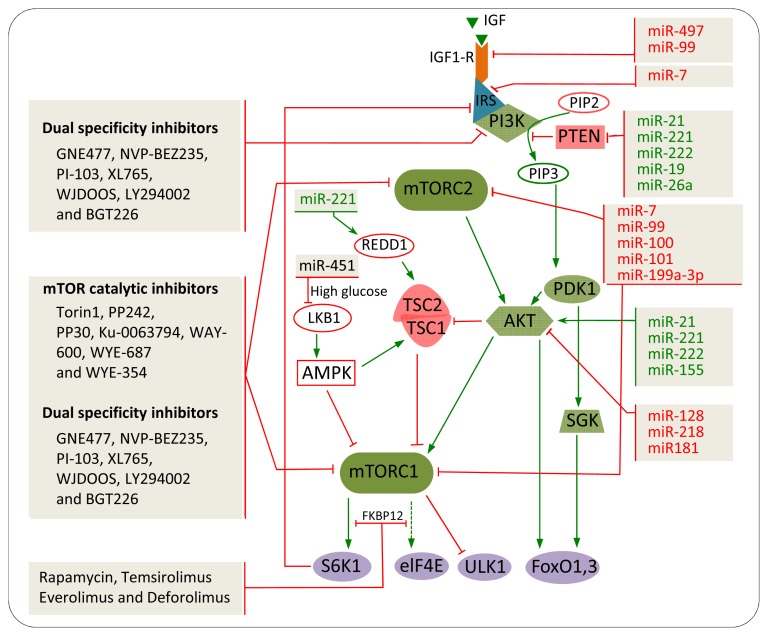
Regulatory networks of mTOR inhibitors and miRNAs in controlling the mTOR pathway in cancer. Green microRNAs (miRNAs) represent oncogenic activities, whereas the red represent tumour suppressor activities. Green lines indicate activation, whereas the red indicate inhibition.

The proposed mechanism by which Rapamycin inhibits mTOR function is through binding to the intracellular receptor FKBP12 at the FRB (FKBP12-rapamycin binding) domain in the C-terminus of mTOR to form FKBP12-rapamycin [21,107]. Subsequently, this inhibitory complex dissociates mTORC1 from its substrates, such as 4E-BPs and S6Ks, or inhibits mTOR kinase activity (Figure 3) [75,108]. Rapamycin and its derivatives (rapalogs) impair mTORC1 function by inhibiting the interaction of raptor with mTOR [108]. mTORC2 is insensitive to acute rapamycin treatment, but chronic treatment interferes with assembly of mTORC2 and inhibits its function [109]. Suppression of cellular proliferation, cell cycle and cell growth by rapalogs has been investigated in many cancers, suggesting that rapalogs could serve as anticancer drugs through mTOR inhibition [110,111]. In human cancer cell lines (neuroblastoma, glioblastoma, small cell lung cancer, prostate cancer, breast cancer and pancreatic cancer), rapalogs inhibit cell proliferation activity [112–116]. In addition, rapalogs have been shown as potent inhibitors of tumour growth in animal tumour models [117]. Furthermore, rapalogs are currently being evaluated in clinical trials for cancer treatment after improved pharmacokinetic properties and reduced immunosuppressive effects were observed [118,119].

Despite the success of rapamycin and its first generation analogs for the treatment of some cancers, such as renal cell carcinoma, the efficacy of these drugs as anti-cancer therapeutics is still limited [120,121]. Analogs of rapamycin inhibit the negative feedback loops from S6K1 to PI3K, leading to upregulation of PI3K signalling, which activates cell proliferation and survival effectors through Akt [122]. Moreover, rapamycin does not inhibit all of mTORC1 functions, as it only partially phosphorylates 4E-BP1 [123,124]. In the case of mTORC2, chronic treatment can only block its function in some type of cancer cell lines, which reflects the inability of rapamycin to inhibit mTORC2 [109].

### 5.2. mTOR Catalytic Inhibitors

mTOR catalytic inhibitors function as ATP-competitive inhibitors of mTOR and inhibit both mTORC1 and mTORC2 functions (Figure 3) [125,126]. These inhibitors include Torin1, PP242, PP30, Ku-0063794, WAY-600, WYE-687 and WYE-354 [126,127]. Compared with rapamycin, mTOR catalytic inhibitors have much greater effects on 4E-BP1 phosphorylation and autophagy [124]. In addition, studies of several cancer cell lines have shown that mTOR catalytic inhibitors suppress protein synthesis and cell proliferation and induce G1 cell cycle arrest [125,127,128]. In animal studies, these drugs showed a better response than rapamycin in leukaemia [129]. Although mTOR catalytic inhibitors have presented better therapeutic response than the first generation rapamycin inhibitors as anticancer drugs, there is a disadvantage. For example, inhibition of S6K1 feedback loop enhances Akt phosphorylation at Thr308 through PDK1 signalling [35].

### 5.3. Dual Specificity Inhibitors

The dual specificity inhibitors, such as GNE477, NVP-BEZ235, PI-103, XL765, WJDOOS, LY294002 and BGT226, disable PI3K, mTORC1, mTORC2, Akt and PDK1 (Figure 3) [130]. This broad inhibition cannot be used to selectively inhibit mTOR [131,132]. However, dual inhibition of PI3K and mTOR was effective as an antiproliferative and antitumor in some cancer cell lines and experimental tumour models [133–135]. For example, NVP-BEZ235 can inhibit the activation of mTORC1, mTORC2, 4E-BP1 and S6 ribosomal protein in breast cancer cells [135]. Furthermore, in a xenograft model of the human breast cancer cell, NVP-BEZ235 inhibited tumour growth [135]. In addition, NVP-BEZ235 inhibited cell migration and cancer metastasis in sarcoma cells [136]. PI-I03, another dual specificity inhibitor, showed antileukaemic effects *in vivo* [129]. Furthermore, PI-I03 has inhibitory effects on tumour growth, invasion and angiogenesis in xenograft models [137].

## 6. miRNAs

miRNAs (microRNA) represent one of a family of noncoding small RNA molecules, which are single-stranded RNAs approximately 22 nucleotides (nt) in length. They post-transcriptionally regulate gene expression by destabilizing translation, suppressing the target mRNA expression and inhibiting translation to regulate multiple biological processes, including cell cycle, differentiation, development and metabolism [138–166]. Over 30% of human genes are regulated by miRNAs [167]. Mature miRNA processing starts with transcription of a long capped and polyadenylated RNA with a hairpin-shaped structure, called the primary transcript (pri-miRNA). Pri-miRNAs are transcribed by RNA polymerase II from non-coding DNA [144]. Subsequently, pri-miRNAs are processed to pre-miRNAs within the nucleus by the RNase III enzyme, Drosha, and Di George syndrome critical region 8 (DGCR8) to create hairpin structures of ~65 nucleotides [145,146]. A nuclear export factor, exportin 5, exports Pre-miRNAs to the cytoplasm [147,148]. In the cytoplasm, the dicing step takes place to produce miRNA duplexes operated by RNase III enzyme, Dicer, TRBP (TAR RNA-binding protein) and Argonaute (AGO), forming the RNA induced silencing complex (RISC), which mediates miRNA activity [149,150]. Following processing, the guide strand remains on the Ago protein, whereas the passenger strand of the duplex is degraded [150]. Mature miRNAs mostly target mRNAs in the 3′-untranslated region (3′-UTR), resulting in mRNA degradation or repressing the translation of mRNA [151]. In addition, some studies have found that miRNAs also target the 5′-UTR and coding sequences of mRNAs and have a role regulating mRNA expression post-transcriptionally [152]. However, there may be other unknown mechanisms involved in miRNA mRNA interactions.

## 7. miRNAs Involved in mTOR Regulation in Cancer

Compelling evidence underscores the ability of a single miRNA to target multiple molecules belonging to the same, as well as to redundant, pathways in neoplastic and normal cells. Thus, understanding the role of miRNAs in tumour cells provides valuable information that will facilitate our understanding of miRNA involvement in the regulation of molecular networks sustaining cancerous phenotypes. To date, evidence shows the involvement of miRNA in cancer initiation, development and progression and identifies this class of regulatory RNAs as diagnostic and prognostic cancer biomarkers, as well as additional therapeutic tools [142]. In addition, growing evidence links miRNA deregulation to carcinogenesis in several human neoplasms, and their molecular signatures of deregulation have been associated with cancer clinical features [143].

miRNAs target the mTOR pathway in several ways, either by interacting directly with mTOR itself or targeting key genes within the pathway, which ultimately affect mTOR function (Figure 3). These genes include upstream regulators of mTOR, such as IGF-R, PI3K and Akt, and negative regulators, such as PTEN (Table 1). miRNAs that are up-regulated in cancer could act as oncogenes and drive tumorigenesis, whereas down-regulated miRNAs act as tumour-suppressors.

### 7.1. IGF-R

IGF is closely interconnected with mTOR since it can regulate mTOR signalling cascades through modulation of the expression of key proteins implicated in the pathway. Recent studies have pointed to a role of miRNAs in regulation of IGF-R in several cancer cells [143–147]. In hepatocellular carcinoma (HCC), miR-99a is inhibited, and restoring its expression suppresses cell growth *in vitro* by activating G1 phase cell cycle arrest [147]. *In vivo,* miR-99a mimics reduced tumour growth and reduced the *α*-fetoprotein level [147]. Both miR-99a and miR-100 are among the most downregulated miRNAs in adrenocortical tumours (ACT), which have been identified to be regulators of IGF-R expression by acting on a target site in the 3′-UTR of its mRNA [143]. In addition, overexpression of miR-497 lead to suppression of IGF1-R activity in colorectal cancer cells [144]. Therefore, downregulation of these miRNAs contributes to elevated activation of the mTOR pathway and malignant behaviour in cancer cells.

### 7.2. PTEN

Loss of PTEN leads to increased PDK1 and AKT due to PIP3 accumulation resulting in activation of mTORC1 and its downstream effectors [70]. Tumour suppressor PTEN is regulated by miRNAs in several cancer cells. Over-expression of the oncogenic miR-221 and miR-222 in non-small cell lung cancer, hepatocarcinoma and gastric cancer cells suppress PTEN expression *in vitro* and *in vivo* experiments leading to activation of the oncoprotein AKT and promotion of cell migration and growth [138,139]. Conversely, knockdown of miR-221 and miR-222 are able to increase PTEN expression and decrease cell migration and invasion [139]. In human squamous cell carcinoma (SCC), oncomir miR-21 targets PTEN and its transcriptional regulator Grainy headlike 3 (Grhl3), leading to activation of mTOR signalling [148]. PTEN is found to be a target of miR-21 in HCC cells, and enhancing miR-21 expression increases tumour cell proliferation, migration and invasion [149]. Moreover, miR-26a is up-regulated in high-grade gliomas and it has been reported as a direct regulator of PTEN [161]. In the *Em-myc* model of mouse B-cell lymphoma, overexpression of miR-19 inhibits PTEN and promotes cell survival via the mTOR pathway [164].

### 7.3. AKT

miRNAs play a key role in targeting Akt by either inducing or suppressing its action. miR-21 is an oncomir that targets Akt by inhibiting Ras homolog gene family member B (Rhob), which leads to activation of cyclin D1 translation in liver regeneration [150,169]. In addition, miR-155 acts as a regulator of proliferation and growth of Waldenström macroglobulinemia cells *in vitro* and *in vivo* by inhibiting AKT phosphorylation [168]. In glioma cells, overexpression of miR-221 and miR-222 increases cell proliferation and invasion *in vitro* and induces glioma growth in xenograft tumour mouse models via activation of Akt [141]. On the other hand, miR-128 acts as a tumour suppressor by increasing Akt phosphorylation, which activates further downstream oncoproteins [165]. Moreover, Akt co-activator T-cell leukaemia/lymphoma 1 (TCL1) is regulated by miR-181 in chronic lymphocytic leukaemia (CCL) [162]. Finally, Epigenetic silencing of miR-218 by overexpression of Rictor in oral squamous cell carcinoma (OSCC) inhibited the phosphorylation of Akt at Ser-473, resulting in cell growth inhibition [166].

### 7.4. mTOR

To understand the role of miRNAs in regulating mTOR, functional studies have been performed *in vitro* and *in vivo* through overexpression or knockdown of miRNAs in cancer cells. For example, miR-199a-3p, miR-100 and miR-7 target the 3′-UTR of mTOR and suppress translation of its mRNA, resulting in activation of the mTOR kinase pathways [147,154,156]. This promotes activation of S6K1 and phosphorylation of 4EBP1 and enhanced protein translation. miR-199a-3p expression is decreased in HCC and restoring its expression level blocks G1-phase cell cycle and impairs invasive capability [156]. Furthermore, in engrafted anaplastic large-cell lymphoma (ALCL) mouse models, miR-101 binds directly to the 3′-UTR of mTOR and leads to reduced tumour growth [155]. Finally, miR-99a expression and mTOR were found to be inversely correlated in human HCC and restored miR-99a expression inhibited cell growth both *in vivo* and *in vitro* [147].

## 8. Combination of miRNAs and Chemical Inhibitors to Target the mTOR Pathway

The mTOR pathway is a complex system, and its deregulation has been implicated in several neoplastic malignancies, Therefore, strategies to inhibit the pathway will most likely include combinatorial approaches. Several studies have looked at the efficacy of combining mTOR inhibitors with miRNAs in cancer cell models. Firstly, suppression of the mTOR pathway by mir-100 enhanced the chemotherapeutic effect of rapamycin analog RAD001 (everolimus) in clear cell ovarian cancer cells [154]. Furthermore, knocking down miRNA-155 expression in low-grade lymphoma, Waldenström macroglobulinemia cells was shown to partially inhibit everolimus-dependent induction of toxicity [170]. This finding indicates that miRNA-155 could be a target responsible for the everolimus-induced anti-Waldenström macroglobulinemia effect [170]. In addition, another rapamycin analogue, CCI-779 (temsirolimus), shows increased anti-proliferative effects in miR-101 negative anaplastic lymphoma kinase (ALK)^+^ large-cell lymphoma (ALCL) cells compared to ALK^−^ cells [155]. When miR-101 is overexpressed in ALCL, cell proliferation weakens in ALK^+^, but not in ALK− cells, and the anti-proliferative effect of temsirolimus on the ALK+ ALCL cells was stronger than ALK− cells, since the effect of miR-101 is partially absent in ALK^−^ cells [155]. One of the reasons that may explain this is the direct binding of miR-101 to the 3′-UTR of mTOR [171].

The strategy of combining both miRNAs and mTOR inhibitors has not been studied extensively in cancer research. In addition, second generation, ATP-competitive inhibitors of mTOR need to be combined with miRNAs and tested to evaluate the potential of using this approach as a chemotherapeutic tool in cancer.

## 9. Conclusion

Over the last decade, the mTOR pathway has received great attention in order to understand its role in cancer cell behaviour. This knowledge has led to the development of therapeutic strategies to target the mTOR pathway. Preclinical studies are currently evaluating their therapeutic potential and they have shown promising anticancer efficacy in certain types of cancers. Rapamycin-based therapeutic approaches showed potent antitumor activity by partially blocking mTORC1 activity in certain types of cancer. In addition, they inhibit the negative feedback loops from S6K1 to PI3K, leading to PI3K-Akt upregulation, which induces cell proliferation and survival effectors. Therefore, the efficacy of these drugs as anti-cancer therapeutics is still limited. mTOR and mTOR-PI3K catalytic inhibitors, such as Torin1, PP242, NVP-BEZ235 and PI-103, have emerged as a new class of mTOR inhibitors targeting both mTORC1 and mTORC2, anticipated to be more effective and have broader applications. However, this kind of inhibition is still in the early stage of evaluation, and its broad therapeutic potential may be toxic to normal cells, especially Dual PI3K-mTOR inhibitors.

Another strategy for targeting the mTOR pathway as a therapeutic tool in cancer is miRNAs. Recently, miRNAs have emerged as modulators of biological pathways that play an essential role in cancer initiation, development and progression through their behaviour as tumour suppressors or oncogenes in several human neoplasms. Current knowledge indicates that miRNAs are involved in regulating the mTOR pathway in some cancer types. Tactics, either by elevating levels of tumour suppressors or inhibiting oncogenic miRNAs, have shown promising therapeutic benefits. However, using miRNAs in targeting the mTOR pathway is still at an early stage. Issues, such as a single miRNA targeting many genes in many pathways, may hinder the potential use of miRNA as therapeutic tools. More *in vivo* studies are needed to clarify the role of miRNAs in cancer and their potential role in enhancing the chemotherapeutic effects of mTOR inhibitors

## Figures and Tables

**Table 1:** miRNA regulatory activities in the mTOR pathway implicated in human cancer.

miRNA	Cancer Cells	Mechanism	Expression	Activity	Biological effects	Reference
*miR-21*	Human squamous cell carcinoma; human hepatocellular cancer (HCC); human glioblastoma (GBM) xenograft model; hepa1, 6 mouse hepatoma	Induced loss of GRHL3 and PTEN expression leading to increased activity of mTOR signalling; targeting PTEN gene through the 3′-UTR binding site and repressing its expression leading to increase activity of AKT and mTOR kinase pathways; downregulation of miR-21 inhibited Akt and affected mTOR activity; activates Akt1/mTORC1-mediated cyclin D1 translation by inhibiting Rhob	Overexpressed	Oncomir	Increased tumour cell proliferation; Promotes cell survival and proliferation; Promotes cell growth; Accelerates hepatocyte proliferation	[142, 148–150]
*miR-7*	Human glioblastoma; human hepatocellular carcinoma (HCC)	Targets IRS; upstream regulator of Akt/mTOR pathway; regulates the expression of mTOR by directly binding to target sites within the 3′-UTR region	Downregulated	Tumour suppressor	Regulates cell invasion	[140,151]
*miR-99*	Human prostate cancer; hepatocellular carcinoma; adrenocortical tumour	Suppresses gene expression by directly binding to the 3′-UTR of IGF-1R and mTOR	Downregulated	Tumour suppressor	Regulates cell growth Induces cell cycle arrest	[143,147, 152]
*miR-221* *miR-222*	Human glioblastomas; Human gastric cancer; Non-small cell lung cancer; Hepatocellular carcinoma	Activates Akt by regulation of common gene expression in gliomagenesis; Regulate PTEN by targeting PTEN; 3′-UTR binding sequences; Enhance cellular migration through blocking PTEN expression leading to activation of the AKT/mTOR pathway; miR-221 targets REDD-1, a regulator of the mTOR kinase signalling	Overexpressed	Oncomir	Induces cell proliferation and cell invasion	[138,139, 141,153]
*miR-100*	Adrenocortical tumour Clear cell ovarian cancer	Interacts with the mTOR 3′-UTR and Raptor genes	Downregulated	Tumour suppressor	Regulation of mitosis and cytokinesis	[143,154]
*miR-101*	Engrafted anaplastic large-cell lymphoma mouse models	Targets mTOR 3′-UTR and suppress mTOR	Downregulated	Tumour suppressor	Reduces tumour growth	[155]
*miR-199*	Hepatocellular carcinoma Osteosarcoma	miR-199a-3p suppresses gene expression by directly binding to the 3′-UTR of mTOR	Downregulated	Tumour suppressor	G1-phase cell cycle arrest Reduces invasive capability Inhibition of cell migration and cell growth	[156,157]
*miR-451*	Glioblastoma	Affects mTOR pathway through AMPK by targeting LKB1, 14-3-3ζ(zeta) and TSC1 in response to glucose	Downregulated	Gene regulator	Regulates cell survival and responsiveness to glucose deprivation	[158–160]
*miR-26*	Murine glioma model	Directly binds to the B2 and B3 sites in the 3′-UTR of PTEN	Overexpressed	Oncomir	Mediates translation and reduces steady-state levels of the protein	[161]
*miR-181*	Lymphocytic Leukaemia	Targets TCL-1 a co-activator of Akt, which enhances Akt kinase activity	Downregulated	Tumour suppressor	Mediates Akt translation	[162,163]
*miR-19*	*Em-myc* model of mouse B-cell lymphoma	Mediates the oncogenic activity miR-17-92 cluster by inhibiting PTEN	Overexpressed	Oncomir	Promotes cell survival	[164]
*miR-497*	Human colon cancer	Downregulation of miR-497 activates mTOR signalling through upregulation of IGF1-R	Downregulated	Tumour suppressor	Suppresses cell proliferation and invasion	[144]
*miR-128*	Glioblastoma	Decreases the Bmi-1 oncogene by binding its 3′-UTR, leading to decreased Akt phosphorylation and reduced mTORC1 activity	Downregulated	Tumour suppressor	Reduces proliferation and self-renewal	[165]
*miR-218*	Oral squamous cell carcinoma	Epigenetic silencing of miR-218 suppresses Akt S473 phosphorylation and reduces Rictor levels	Downregulated	Tumour suppressor	Induces growth inhibition	[166]
*miR-155*	Waldenström macroglobulinemia	Regulates mTOR by AKT phosphorylation	Overexpressed	Oncomir	Regulates cell proliferation cell-cycle, migration and adhesion	[168]
